# New rhodamine B-based chromo-fluorogenic probes for highly selective detection of aluminium(iii) ions and their application in living cell imaging†

**DOI:** 10.1039/c8ra09850f

**Published:** 2019-02-19

**Authors:** Xin Leng, Wenfeng Xu, Chengfang Qiao, Xu Jia, Ying Long, Bingqin Yang

**Affiliations:** Key Laboratory of Synthetic and Natural Functional Molecule Chemistry of Ministry of Education, College of Chemistry and Materials Science, Northwest University Xi'an 710127 China organic_lengxin@163.com; Shaanxi Key Laboratory of Comprehensive Utilization of Tailings Resources, College of Chemical Engineering and Modern Materials, Shangluo University Shangluo 726000 China xiaoqiaoqcf@126.com

## Abstract

Two rhodamine B-based fluorescent probes, BOS1 and BOS2, were designed and synthesized with good yields *via* the condensation reactions between the *o*-diaminobenzene modified rhodamine core structure (RBO) and salicylaldehyde derivatives. Both the probes exhibited remarkable absorbance-on and fluorescence-on responses to Al^3+^ over other metal ions in ethanol–water (1 : 9, v/v) medium *via* the rhodamine ring-opening approach, which can be used for “naked-eye” Al^3+^ detection over a broad pH range (5–9). The fluorescence intensities of the probes were linear with the Al^3+^ ion concentration, resulting in a low limit of detection of 1.839 μM (BOS1) and 1.374 μM (BOS2) for Al^3+^. In addition, the MTT assays and cell imaging experiments of Al^3+^ in SGC-7901 living cells demonstrated that the probes had negligible cytotoxicity, and were cell permeable and suitable for sensing Al^3+^ in biological systems.

## Abstract 2

Two rhodamine B-based fluorescent probes exhibited remarkable absorbance-on and fluorescence-on responses to Al^3+^, which can be used for biological imaging.

## Graphical abstract

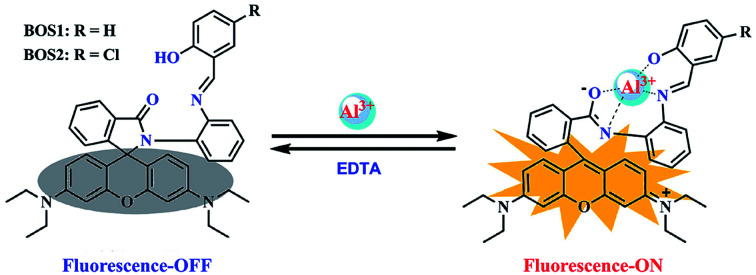

## Introduction

As the most abundant metal element in the earth's crust, aluminium has been widely used in our daily life, such as in food additives, clinical drugs, kitchen utensils, packing materials, water treatment, *etc.*^1–5^ However, the heavy and inappropriate use of aluminium products in recent years has created adverse effects on life and environmental systems due to its toxicity.^6–8^ In particular, the excessive intake of dissolved Al^3+^ easily causes accumulation in the human body, which may cause several disorders, including Alzheimer's disease, osteomalacia, Parkinson's disease, and breast cancer.^9–12^ Therefore, the fast detection and quantitative analysis of Al^3+^ in the human body are crucially important for health warnings.

Because of the insufficient spectroscopic characteristics of Al^3+^, the reported Al^3+^-detection techniques, such as ^27^Al NMR technology, atomic absorption spectrometry (AAS),^13^ inductively coupled plasma atomic emission spectrometry (ICP-AES),^14^ mass spectrometry and electrochemical methods,^15^ are usually complex, time-consuming and costly. Fluorescence analysis, as a type of highly selective and sensitive detection method, not only possesses the features of easy operation, low cost, low detection limit and rapid response, but also can be used for real-time living organism detection, has attracted significant interest of researchers.^16–20^ Apparently, the reasonable design and preparation of effective biocompatible Al^3+^ fluorescent chemosensors are urgent problems need to be solved currently.^21–24^

Considering the weak coordination ability and strong tendency to hydrolysis of Al^3+^ ions, small-molecular fluorescent probes with bright chromophore and multidentate coordination sites are highly promising. Recent studies reveal that the rhodamine-based probes are regarded as ideal candidates for the “off–on” fluorescent sensors owing to the excellent photophysical properties such as remarkable photostability, high fluorescence quantum yields, special response mechanism, strong anti-interference ability and long emission wavelength.^25–28^ On one hand, with the introduction of target metal ions, the rhodamine framework can display “turn-on” fluorescence signals through structural change between the spirocyclic and open-cycle forms.^29^ On the other hand, the free-rotating benzoic acid group can be accessibly substituted by the functional groups with multidentate chelation sites to chelate target ions for fluorescence sensor without changing the spirolactam forms.^30^ Particularly, the Schiff base rhodamine probes have received lots of attention due to their high sensitivity and selectivity, rapid response time and ease of synthesis,^31,32^ where the imines functional groups can be generated by the introduction of different carbonyl or amines to adjust the spectral response range of the rhodamine matrix, and at the same time to form suitable “ONO”, “ONN” or “ONNO” sites to rapidly anchor the target metal ions.^33^ Moreover, the imines also provide the opportunity of C=N isomerization for probes to generate richer fluorescence sensing pathway.^34^

**Scheme 1:**
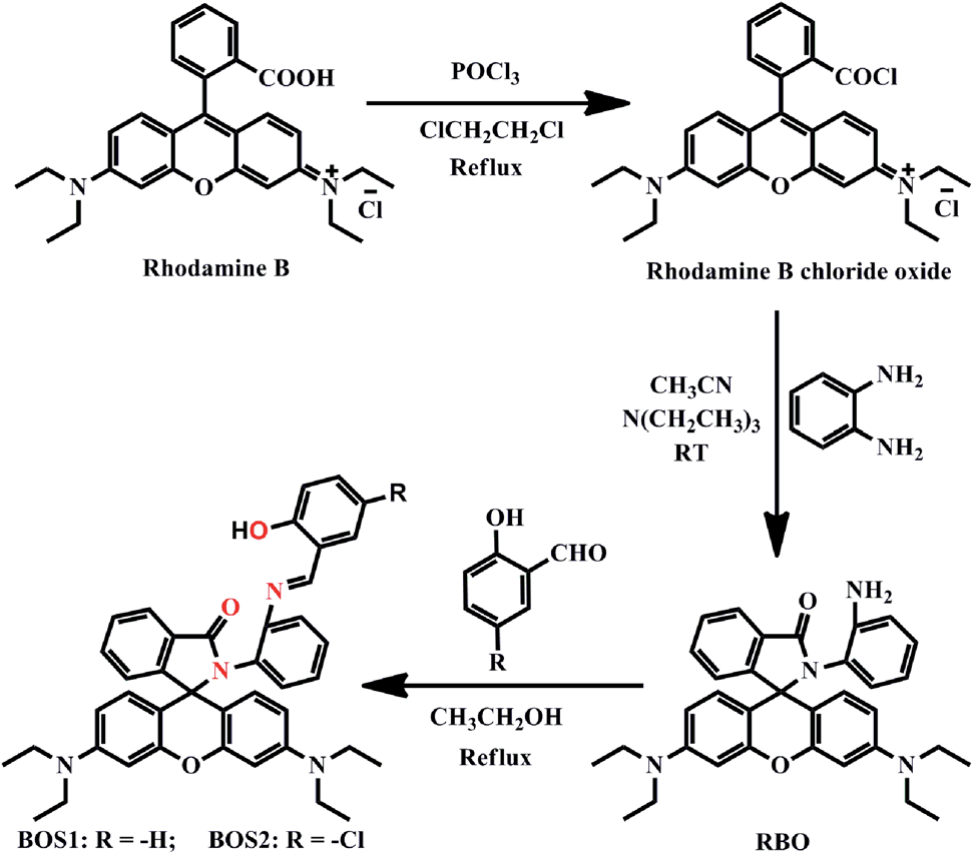
Syntheses of BOS1 and BOS2.

Herein, two new rhodamine B-based sensors, BOS1 and BOS2 were designed and prepared by a three-step synthesis with simple raw materials (Scheme 1). The two chemosensors show highly selective and sensitive fluorescence response to Al^3+^ with the ring-opening sensor mechanisms described above, which can be used for naked-eye detection with rapid switch-on fluorescence and remarkable color changes. Furthermore, these advanced characteristics endow the two probes highly promising for biological imaging applications.

## Experimental

### Materials and methods

All chemicals were of analytical-reagent grade, and they were commercially available from commercial sources and used without further purification. The SGC-7901 living cells (human gastric carcinoma cells) were obtained from Xi'an Jiaotong University Health Science Center. The twice-distilled water was used throughout the experiment. The solid powders of probes BOS1/BOS2 were dissolved in ethanol solution in concentration of 1 mM as stock solutions. And then took out quantificational BOS1/BOS2 in different testing systems. Fluorescence spectra were carried on a HITACHI F-4500 fluorescence spectrophotometer. UV-vis spectra were performed on a Shimadzu UV-1700 spectrophotometer. The elemental analyses of C, H, and N were performed on a Vario EL III elemental analyzer. IR spectra were recorded on a Bruker Tensor 27 spectrometer. NMR spectra were obtained on a Varian INOVA-400 MHz spectrometer (at 100 MHz for ^13^C NMR and 400 MHz for ^1^H NMR). A Bruker micro TOF-Q II ESI-TOF LC/MS/MS Spectroscopy was used to perform mass spectra. Melting point tests were taken on an XT-4 micromelting apparatus and uncorrected. Results of cytotoxicity were analyzed with the Soft max pro software (version 2.2.1) in Spectra max190-Molecular Devices. The living cells imaging were performed on an Olympus FV1000 confocal microscopy with *λ*_ex_ = 400 nm.

### Synthetic procedures

#### Synthesis of RBO

Phosphorus oxychloride (1 mL, 10.09 mmol) was added to a dry 1,2-dichloroethane solution (20 mL) of rhodamine B (1.59 g, 3 mmol), and the resulting mixture was refluxed for 6 h. After cooling to room temperature and removal of the solvent *in vacuo*, the crude product of rhodamine B chloride oxide was obtained without any purification. Then the residue was directly reacted with an acetonitrile solution (30 mL) of *o*-phenylenediamine (0.324 g, 3 mmol) at room temperature for 30 min, followed by addition of 2 mL triethylamine as acid-capturer, the mixture was stirred for 8 h. After removal of the solvent under reduced pressure, the crude product was purified by silica gel column chromatography to give RBO in a yield of 89.3%. HRMS (ESI) calcd for (C_34_H_36_N_4_O_2_) *m*/*z* = 532.2838, found: 533.2897 (M + H)^+^. ^1^H NMR (400 MHz, CDCl_3_), *δ*: 8.07–8.09 (d, *J* = 8 Hz, 1H), 7.57–7.62 (q, *J* = 8 Hz, 2H), 7.29–7.31 (d, *J* = 8 Hz, 1H), 6.98–7.01 (t, *J* = 4 Hz, 1H), 6.80–6.08 (m, 9H), 3.32–3.37 (dd, *J* = 8 Hz, 8H), 1.17–1.21 (t, *J* = 8 Hz, 12H).

#### Synthesis of BOS1

RBO (0.53 g, 1 mmol) was dissolved in 20 mL ethanol. Then a solution of salicylaldehyde (0.12 g, 1 mmol) in ethanol (20 mL) was added and the mixture was stirred and refluxed for 4 h at 80 °C. After removal of the solvent under reduced pressure, the resulting precipitate was purified by silica gel column chromatography to give BOS1 (light yellow power) in 76.3% yield. Mp: 158–159 °C. Anal. calcd for C_41_H_40_N_4_O_3_ (%): C, 77.33; H, 6.33; N, 8.80. Found: C, 77.28; H, 6.36; N, 8.91. HRMS (ESI) calcd for (C_41_H_40_N_4_O_3_) *m*/*z* = 636.3100, found: 659.2972 (M + Na)^+^. IR (KBr) *ν*: 3444, 2968, 1699, 1616, 1514, 1356, 1223, 1115, 754, 638 cm^−1^. ^1^H NMR (400 MHz, CDCl_3_) *δ* (ppm): 12.50 (s, 1H), 8.12–5.91 (m, 18H), 5.59–5.46 (d, *J* = 8 Hz, 1H), 3.39–3.20 (dq, *J* = 8 Hz, 4H), 3.17–2.90 (d, *J* = 8 Hz, 4H), 1.23–1.16 (t, *J* = 8 Hz, 6H), 1.13–0.96 (t, *J* = 8 Hz, 6H). ^13^C NMR (100 MHz, CDCl_3_), *δ* (ppm): 166.1, 163.0, 161.1, 154.7, 153.4, 151.7, 149.1, 148.5, 147.8, 132.8, 132.5, 132.4, 129.8, 129.1, 127.1, 124.0, 120.6, 119.7, 118.4, 117.3, 108.3, 107.4, 106.3, 97.9, 97.0, 66.1, 44.6, 44.1, 12.6.

#### Synthesis of BOS2

RBO (0.53 g, 1 mmol) was dissolved in 20 mL ethanol. Then a solution of 5-chlorosalicylaldehyde (0.16 g, 1 mmol) in ethanol (20 mL) was added and the mixture was stirred and refluxed for 2 h at 80 °C. After removal of the solvent under reduced pressure, the resulting precipitate was purified by silica gel column chromatography to give BOS2 (light yellow power) in 78.5% yield. Mp: 154–156 °C. Anal. calcd for C_41_H_39_N_4_O_3_Cl (%): C, 73.36; H, 5.86; N, 8.35. Found: C, 73.04; H, 5.98; N, 8.24. HRMS (ESI) calcd for (C_41_H_39_N_4_O_3_Cl) *m*/*z* = 670.2711, found: 671.2773 (M + H)^+^. IR (KBr) *ν*: 3442, 2968, 1699, 1616, 1514, 1351, 1223, 1116, 756 cm^−1^. ^1^H NMR (400 MHz, CDCl_3_), *δ*: 12.53 (s, 1H), 8.48–5.80 (m, 17H), 5.55 (s, 1H), 3.44–3.23 (m, 4H), 3.11 (s, 4H), 1.23–0.98 (m, 12H). ^13^C NMR (100 MHz, CDCl_3_) *δ*: 166.1, 161.3, 159.7, 154.7, 153.6, 151.4, 149.2, 148.4, 147.8, 132.5, 131.1, 130.4, 129.8, 129.0, 127.1, 123.9, 120.6, 119.5, 118.9, 117.3, 108.4, 107.5, 106.6, 98.0, 97.0, 68.2, 44.6, 44.1, 12.6.

### Preparation of the test solution

The 10 μM stock solution of probes BOS1/BOS2 were prepared in ethanol–water (1 : 9, v/v, Tris–HCl, pH = 7.2). The solutions of various testing cation species were prepared from Ca(NO_3_)_2_·4H_2_O, Mg(NO_3_)_2_·6H_2_O, Co(NO_3_)_2_·6H_2_O, AgNO_3_, Cd(NO_3_)_2_·4H_2_O, Zn(NO_3_)_2_·6H_2_O, Cu(NO_3_)_2_·3H_2_O, KNO_3_, NaNO_3_, LiNO_3_, Mn(NO_3_)_2_·4H_2_O, Pd(NO_3_)_2_·2H_2_O, Hg(NO_3_)_2_·H_2_O, Ni(NO_3_)_2_·6H_2_O, Sn(NO_3_)_2_, Pb(NO_3_)_2_, Cr(NO_3_)_3_·9H_2_O, Ba(NO_3_)_2_, Al(NO_3_)_3_·9H_2_O, and Fe(NO_3_)_3_·9H_2_O dissolved in the double distilled water. Before spectroscopic measurements, the corresponding solutions of probes were freshly prepared by diluting the high concentration stock solutions. All the measurements were made according to the procedures as follows. Placing 1 mL of the probe solution and an appropriate aliquot of each metal stock into a 10 mL glass tube, and diluting the solution to 10 mL with ethanol–water (1 : 9, v/v, Tris–HCl, pH = 7.2) solution.

### Cytotoxicity assays

The MTT assays were performed to evaluate the toxicity of BOS1, BOS2 and Al^3+^ by SGC-7901 living cells.^35^ 90% confluent cells were chosen, digested by 1 mL 0.25% of trypsin, and transferred in 96-well plates. The cells were treated and incubated at 37 °C under 5% CO_2_ in culture medium (DMEM (Dulbecco's Modified Eagle Medium) + 10% FBS (Fetal Bovine Serum)) and maintained 24 h. Different concentrations of BOS1, BOS2 and Al^3+^ were added to the 96-well plates, respectively. Another 24 hours incubation was taken at the same condition. Following this, the medium was removed and washed three times with phosphate buffered saline (PBS). Then the medium was replaced with mixed liquor of MTT (5 mg mL^−1^) and culture medium, and incubated for an additional 4 h. After that, the MTT was removed and washed three times with PBS. Subsequently, 150 μL DMSO was carefully added to each well and ultrasonic oscillation for 10 minutes. All the experiments were conducted in triplicate. The cell viability (%) was calculated according to the equation: cell viability (%) = [OD_490_ (sample)/OD_490_ (control)] × 100%, where OD_490_ (sample) represents the optical density of the wells treated with various concentration of probes or metal ions and OD_490_ (control) represents that of the wells treated with ethanol.

### Cell culture and fluorescence imaging

The SGC-7901 living cells (human gastric carcinoma cells) were cultured in DMEM replenished with 10% FBS. Before the experiments, cells were processed with probes BOS1/BOS2 (20 μM) for 1 h at 37 °C in humidified air and 5% CO_2_, washed three times with PBS then imaged. After incubation with Al^3+^ (20 μM) for another 1.5 h at 37 °C, cells were washed three times with PBS to remove remaining metal ions and then imaged. Confocal fluorescence imaging was carried out with an Olympus FV1000 laser scanning microscope with 80× objective lens.

## Results and discussion

### Spectroscopic properties

Both of the probes BOS1 and BOS2 exhibited highly selective and sensitive response to Al^3+^ ion in ethanol–water (1 : 9, v/v). Although the different salicylaldehyde moieties had effects on the fluorescence/absorption intensities and response time, no obvious differences were observed on the other spectral properties of the two probes. Therefore, only the property of BOS1 is described in detail. The optical spectra of BOS2 are given in the ESI.†

**Fig. 1:**
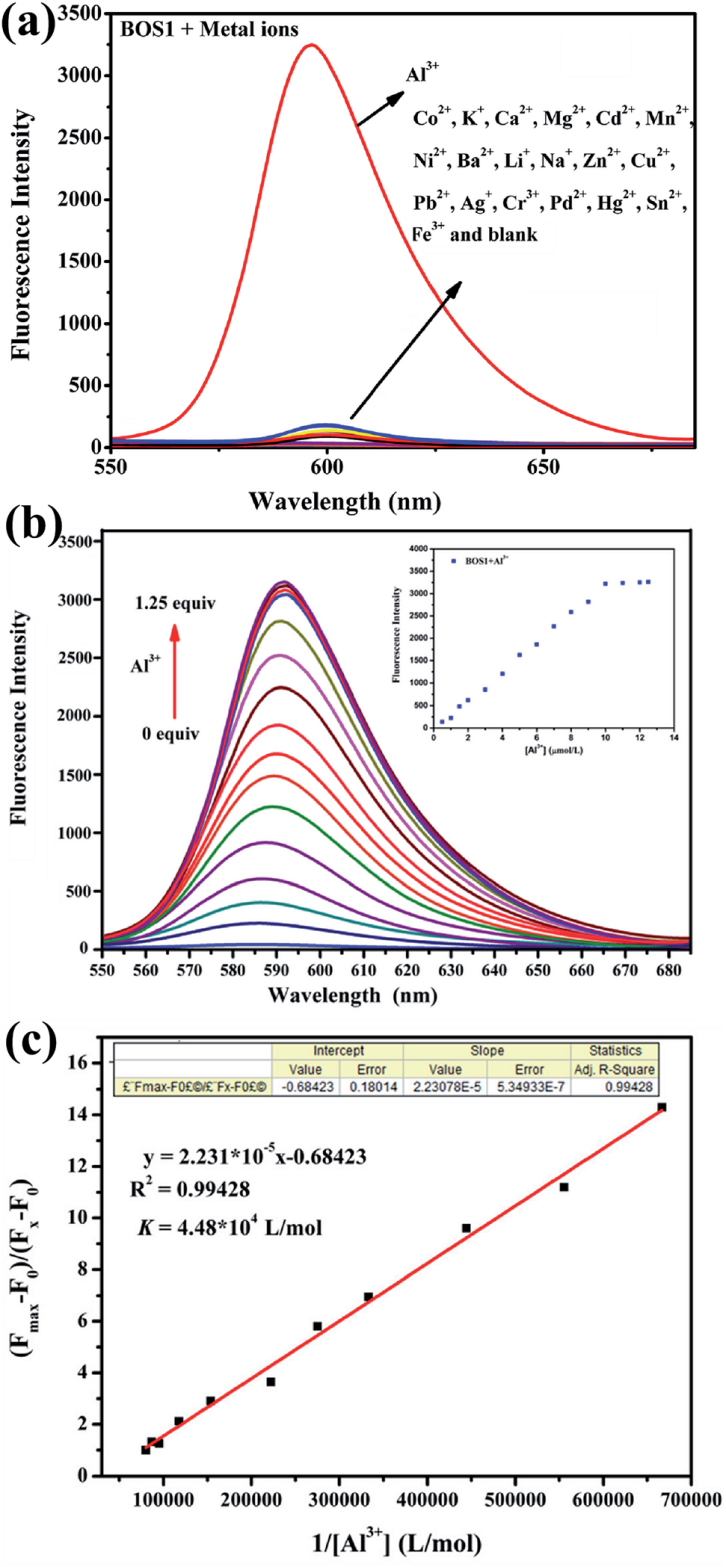
(a) Fluorescence spectra of BOS1 (10 μM) in ethanol–water (1 : 9, v/v, Tris–HCl, pH = 7.2) solution upon addition of various metal ions (10 μM). (b) Fluorescence spectra of BOS1 (10 μM) with the addition of various concentrations of Al^3+^ ions (0–12.5 μM) in ethanol–water (1 : 9, v/v, Tris–HCl, pH = 7.2) solution. Inset: changes in the emission intensity at 592 nm. (c) Determination of binding constant of BOS1 with Al^3+^ using Benesi–Hildebrand equation.

The fluorescence sensing ability of BOS1 toward metal ions was investigated in ethanol–water (1 : 9, v/v, Tris–HCl, pH = 7.2) solution. As shown in Fig. 1a, the free BOS1 exhibited negligible fluorescence emission due to its spirolactam form. Upon the further addition of different metal ions (Co^2+^, K^+^, Ca^2+^, Mg^2+^, Cd^2+^, Mn^2+^, Ni^2+^, Ba^2+^, Li^+^, Na^+^, Zn^2+^, Cu^2+^, Pb^2+^, Ag^+^, Cr^3+^, Pd^2+^, Hg^2+^, Sn^2+^, Fe^3+^, Al^3+^), only the Al^3+^ ion led to a remarkable luminescence enhancement at a maximum emission wavelength of 592 nm, while no obvious luminescent intensity changes were observed in the presence of other metal ions. These results indicated the high selectivity of the BOS1 probe for Al^3+^ detection.

**Fig. 2:**
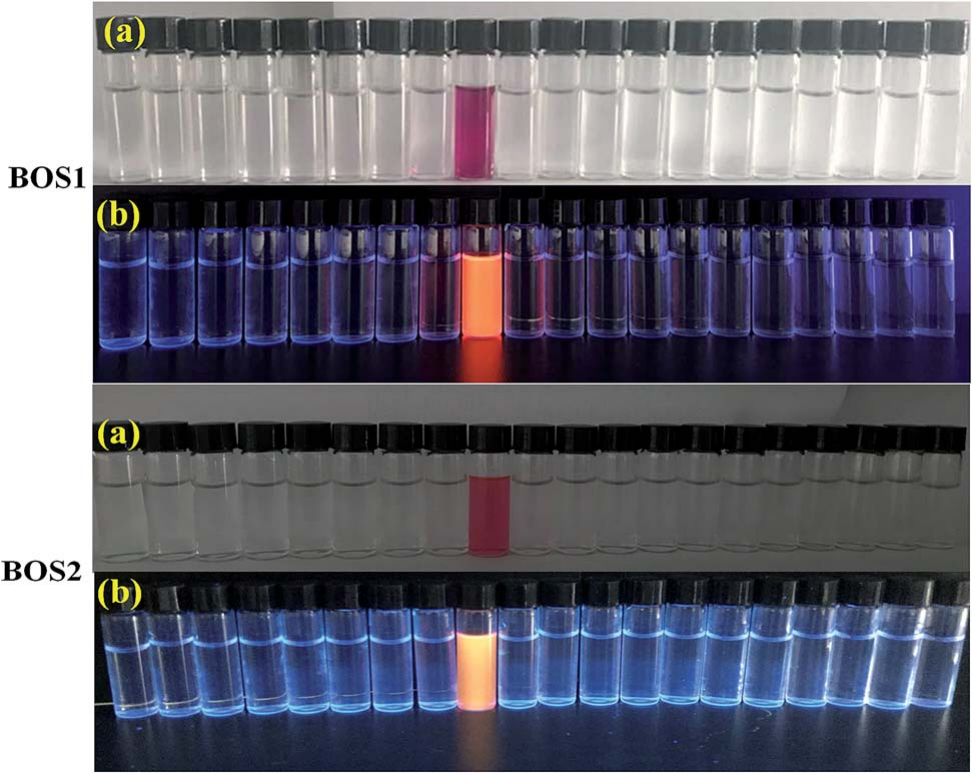
Pictures of BOS1 and BOS2 as selective naked-eye chemosensors (a) and the visual fluorescence emissions by using a UV lamp (365 nm) (b) for Al^3+^. From the left to right: Li^+^, Na^+^, K^+^, Ag^+^, Mg^2+^, Ca^2+^, Ba^2+^, Fe^3+^, Al^3+^, Cr^3+^, Sn^2+^, Co^2+^, Cd^2+^, Mn^2+^, Ni^2+^, Zn^2+^, Cu^2+^, Pb^2+^, Pd^2+^, Hg^2+^.

Fluorescence titration experiments were performed to investigate the interaction between BOS1 and Al^3+^ (Fig. 1b). Since the stable and characteristic “spirolactam form” of rhodamine B group, free BOS1 shows colorless and no fluorescence response in the visible region range from 480 to 660 nm. However, along with the gradual addition of Al^3+^, the fluorescence emission intensity at 592 nm was significantly enhanced with a color variation from colorless to orange (Fig. 2), suggesting that the xanthene moiety of rhodamine B was subjected to the delocalization interference, and BOS1 was a true “off–on” chemosensor for Al^3+^. After the addition of 1.0 equiv. of Al^3+^, the titration curve reached a steady plateau accompanied by more than 100-fold increase in the emission at 592 nm compared with that of free BOS1. Such significant enhancement of fluorescence clearly indicated that the spirolactam form of rhodamine B was unfolded or the rotation of the “C=N” group were inhibited owing to gradually adding Al^3+^ to BOS1, and a highly delocalized π-conjugated system was ultimately formed. The association constant *K*, of BOS1 with Al^3+^ was calculated according to the Benesi–Hildebrand equation:^36,37^(*F*_max_ − *F*_0_)/(*F*_x_ − *F*_0_) = 1 + (1/*K*)(1/[Al^3+^]),where *F*_max_, *F*_0_ and *F*_x_ are the fluorescence intensities of probe in the presence of Al^3+^ at saturation, free probe, and any intermediate Al^3+^ concentration, respectively. The binding constant value was found to be *K* = 4.48 × 10^−4^ M (for BOS2: *K* = 4.92 × 10^−4^ M, Fig. S3†).

**Fig. 3:**
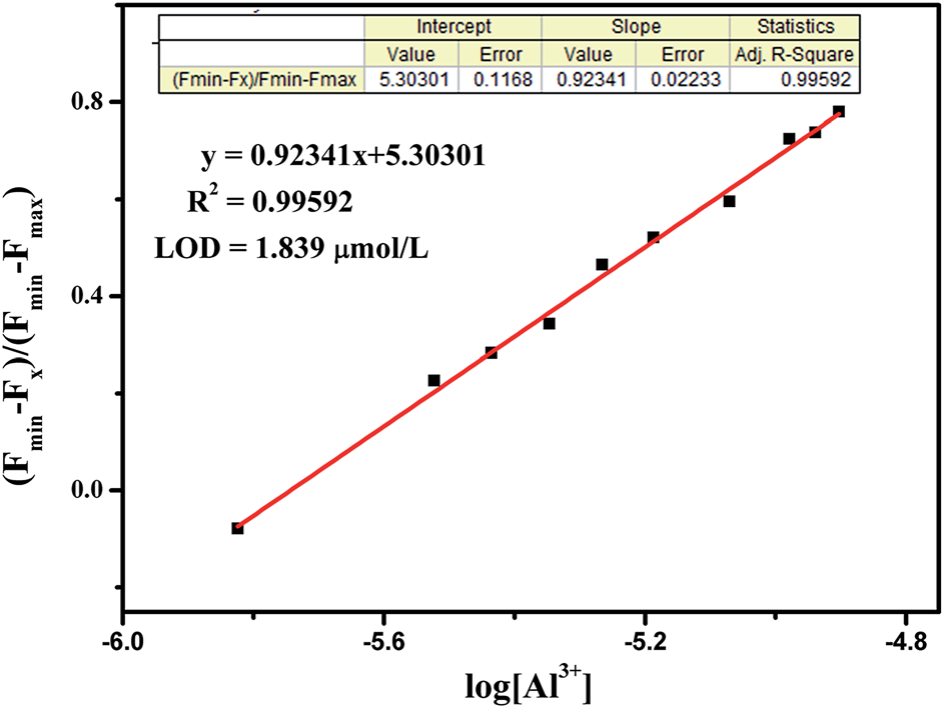
The plot of (*F*_min_ − *F*_x_)/(*F*_min_ − *F*_max_) *versus* log[Al^3+^] for the probe BOS1.

Moreover, the detection limit of BOS1 and BOS2 were determined from the result of titrating experiment. As shown in Fig. 3, according to the widely used method,^38,39^ linear regression curves were fitted based on the plots of (*F*_min_ − *F*_x_)/(*F*_min_ − *F*_max_) *vs.* log[Al^3+^], where the *F*_x_ is the fluorescence intensity at 592 nm at each concentration of Al^3+^ added, *F*_min_ and *F*_max_ are respectively the minimum and maximum fluorescence intensity at 592 nm, thus the intercepts of the lines at *x*-axis were taken as the detection limit of BOS1 (1.839 μM) and BOS2 (1.374 μM) (Fig. S4†).

**Fig. 4:**
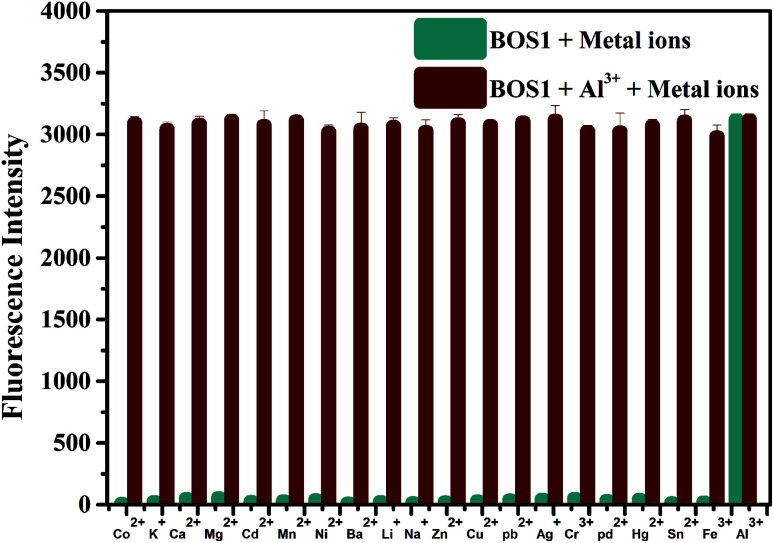
Fluorescence intensity changes of BOS1 (10 μM) upon the addition of various metal ions (10 μM) in the presence of Al^3+^ (10 μM) in ethanol–water (1 : 9, v/v, Tris–HCl, pH = 7.2) solution. The teal bars represent the fluorescence response of BOS1 and metal ions. The dark red bars represent the subsequent addition of 10 μM Al^3+^ to the above solutions.

The outstanding selectivity to the target detective metal ion over other potentially competing species is crucial for the application of metal ions sensors. So the fluorescence response of BOS1 (10 μM) towards Al^3+^ ions and other various metal ions in EtOH/H_2_O = 1/9 (v/v, Tris–HCl, pH = 7.2) were investigated. As depicted in Fig. 4, no obvious changes of fluorescence intensity could be detected when 1.0 equiv. metal ions (Co^2+^, K^+^, Ca^2+^, Mg^2+^, Cd^2+^, Mn^2+^, Ni^2+^, Ba^2+^, Li^+^, Na^+^, Zn^2+^, Cu^2+^, Pb^2+^, Ag^+^, Cr^3+^, Pd^2+^, Hg^2+^, Sn^2+^, Fe^3+^) were added into the relevant solution. Conversely, about 100-fold enhancement of emission intensity at 592 nm appeared obviously in the presence of subsequent 1.0 equiv. Al^3+^ ions, indicating that the recognition of Al^3+^ ions by the probe BOS1 is not interfered by other coexisting metal ions. The above facts reveal that the BOS1 shows high selectivity, anti-interference and sensitivity toward Al^3+^ ions, and could be potentially applied to detect Al^3+^ ions in complex systems. The similar Al^3+^ response performances were also observed for the probe BOS2 (Fig. S5†).

**Fig. 5:**
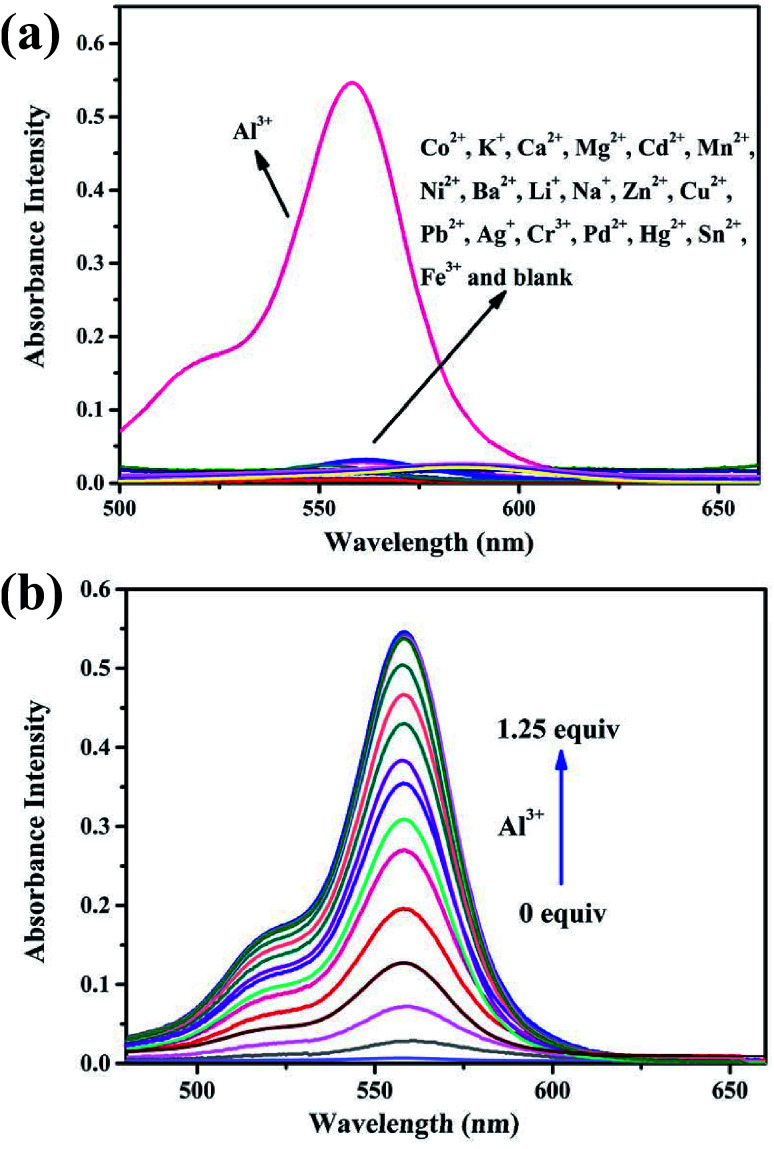
(a) UV-vis absorption spectra of BOS1 (10 μM) in ethanol–water (1 : 9, v/v, Tris–HCl, pH = 7.2) solution upon addition of various metal ions (10 μM). (b) UV-vis absorption spectra of BOS1 (10 μM) with the addition of various concentrations of Al^3+^ ions (0–12.5 μM) in ethanol–water (1 : 9, v/v, Tris–HCl, pH = 7.2) solution.

The UV-vis absorption of the probes (10 μM) was also investigated in an ethanol–water (1 : 9, v/v, Tris–HCl, pH = 7.2) solution. As can be seen in Fig. 5a and S6,† both the free BOS1 and BOS2 exhibited nearly no absorption bands in the visible region, which may be attributed to their closed spirolactam forms. As expected, a significant enhancement of the absorption at 568 nm (BOS1) or at 559 nm (BOS2) was observed in the presence of Al^3+^ ions, whereas tiny absorption changes occurred when nineteen other metal ions added, respectively. With the increasing concentration of Al^3+^ ions in the range of 0–100 μM, the absorption band gradually enhanced, indicating the ring-opening form of the rhodamine spirolactam of BOSs. The absorption intensity showed negligible changes with further increasing the concentration of Al^3+^ (up to 12.5 μM), which suggested the saturated binding behaviours between Al^3+^ and BOS probes (Fig. 5b and S7†). The association constant for Al^3+^ ions was calculated to be 4.39 × 10^4^ M^−1^ (BOS1) and 4.76 × 10^4^ M^−1^ (BOS2) from the absorption titration curves. Moreover, the dramatical color changes from colorless to peach-red associated with the reaction of probes with Al^3+^ ions were visually detectable with good selectivity, which indicated that BOS1/BOS2 could successfully serve as “naked-eye” probes for Al^3+^ detection (Fig. 2).

**Fig. 6:**
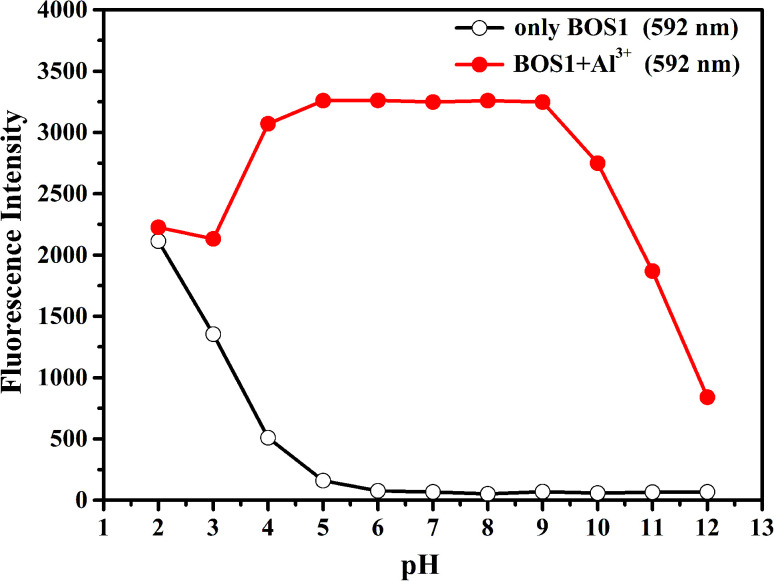
Effects of pH on BOS1 (10 μM) response to Al^3+^ (the pH of solution was adjusted by aqueous solution of NaOH (1 M) and HCl (1 M)).

It is well known that the spirolactam ring of the rhodamine derivative is commonly open in acidic media and shows the fluorescence of rhodamine. Therefore, the optimal pH conditions for the probes BOS1/BOS2 should be evaluated to affirm their stabilities for potential practical applications. The pH dependent fluorescence responses of BOS1 and BOS2 in the presence and absence of Al^3+^ were recorded in the pH range of 2–12 (Fig. 6 and S9†). For BOS1 system, the fluorescence intensities of both BOS1 and the BOS1–Al^3+^ species were strong enough when pH < 4, which could be due to the ring opening of rhodamine derivatives induced by strong protonation of the tertiary amine-N atom in acid conditions. No obvious emission of free BOS1 was observed when pH > 5, while the strong fluorescence emissions after the addition of Al^3+^ within the pH range of 5–9 were detected, which revealed that the BOS1–Al^3+^ complex was formed in this pH region and the BOS1 probe towards Al^3+^ could work well in such approximate physiological conditions with a low background response. With further increasing the pH value, the emission intensities were quenched because of the decoordination of Al^3+^, leading to the formation of Al(OH)_3_ and the reformation of spirolactam rings. Similar events were also found in the BOS2 system, the BOS2–Al^3+^ species presented the strongest fluorescent responses within an optimal pH range of 5–9. The suitable pH response range suggested that no buffer solutions were required for the detection of Al^3+^, and both BOS1 and BOS2 would provide the potential practical applications in environmental systems or living cells.

**Fig. 7:**
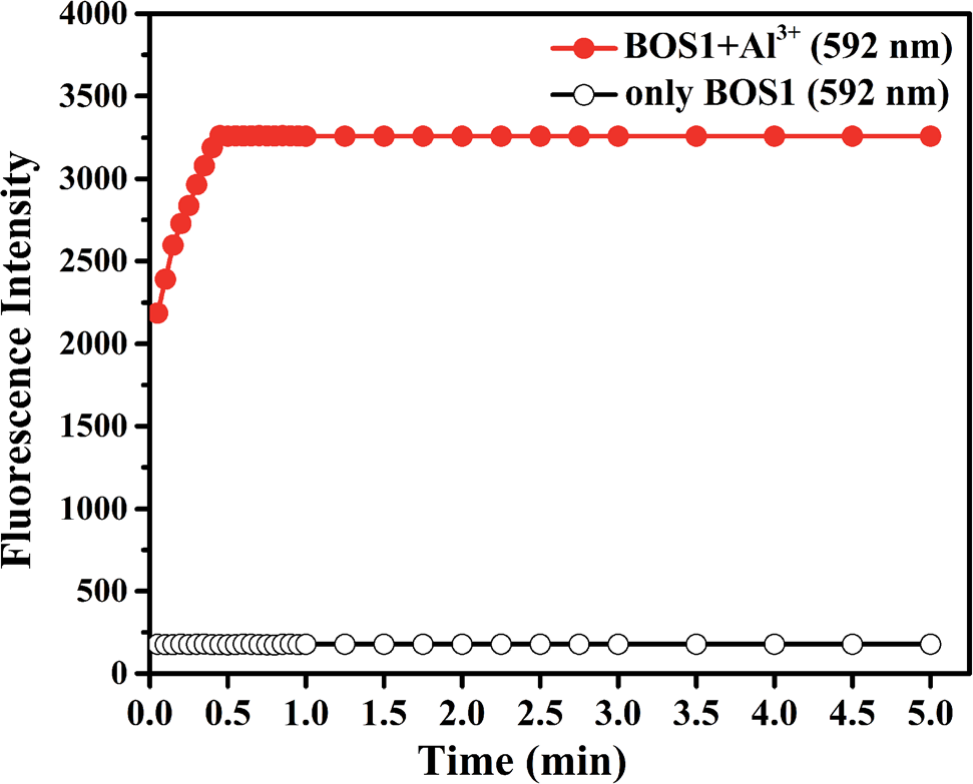
Effects of time on BOS1 (10 μM) response to Al^3+^ in ethanol–water (1 : 9, v/v, Tris–HCl, pH = 7.2) solutions.

In addition, the response time is important to the application of naked-eye detection. So the time dependent fluorescence responses of BOS1 and BOS2 in the presence of Al^3+^ were carried out in a simulated *in vivo* environment (ethanol–water 1 : 9, v/v, Tris–HCl, pH = 7.2) at room temperature. As the BOS1/BOS2 interacted with Al^3+^, the fluorescence intensities of the analysis systems significantly increased to the maximum value within approximately 30 s for BOS1 (Fig. 7) and 42 s for BOS2 (Fig. S10†). These results show that BOS1 and BOS2 are reliable instantaneously responsive colorimetric sensor for Al^3+^.

### Proposed mechanism for the interactions between probes and Al^3+^

**Scheme 2:**
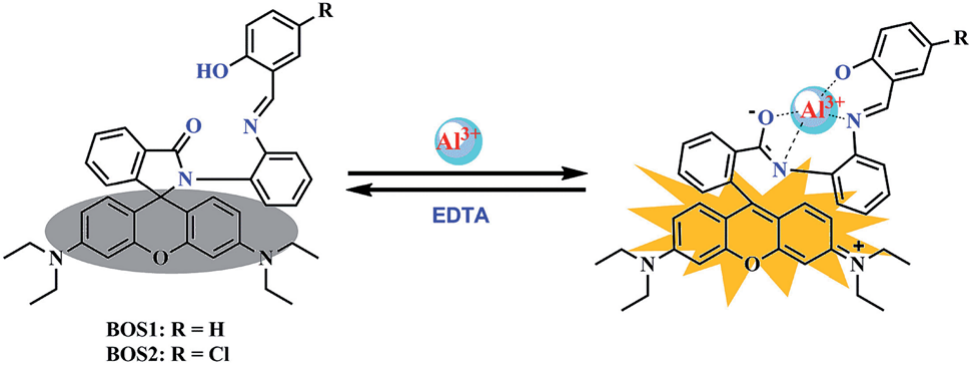
Proposed mechanisms of the interactions between BOS1/BOS2 and Al^3+^ ions.

According to the results of spectroscopic responses of BOS1/BOS2 to Al^3+^, we speculated that the probable binding ways and interaction mechanisms between BOS1/BOS2 and Al^3+^ were likely due to the chelation-induced ring opening of rhodamine spirolactam (Scheme 2), rather than other possible reactions, which were similar to those reported in previously literatures.^40–44^ That is, the Al^3+^ ions coordinated to the phenolic hydroxyl O, imino N, benzoylamide O and N atoms of the probes to form conjugated moieties, and the lactam rings of rhodamine were induced to be opened, exhibiting significant fluorescence enhancements.

**Fig. 8:**
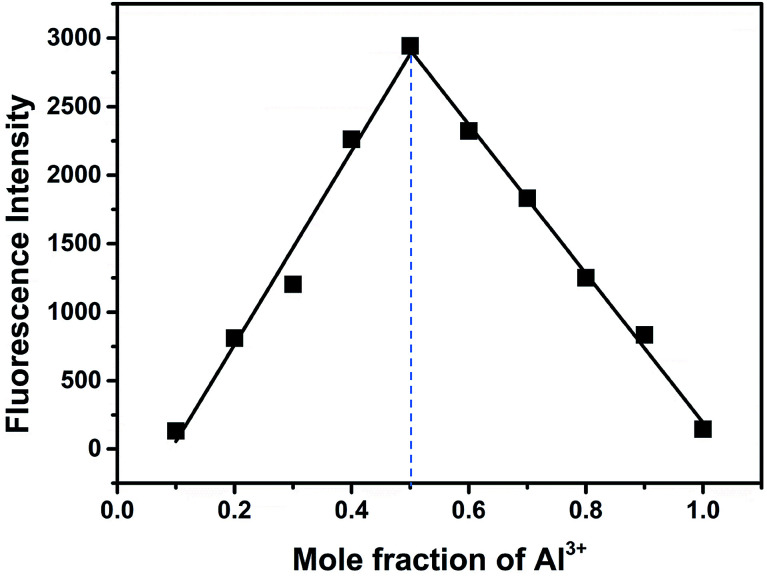
Job's plot of BOS1 and Al^3+^ (the total concentration was 10 μM).

Binding analysis was further performed to determine the ratio between probes and Al^3+^ by using the method of continuous variations (Job's plots). As shown in Fig. 8, a maximum fluorescence emission at 592 nm was observed when the molecular fraction of Al^3+^ is close to 0.5, which revealed that the Al^3+^-chemodosimeter displayed 1 : 1 stoichiometry, and further proved the above-mentioned binding modes.

**Fig. 9:**
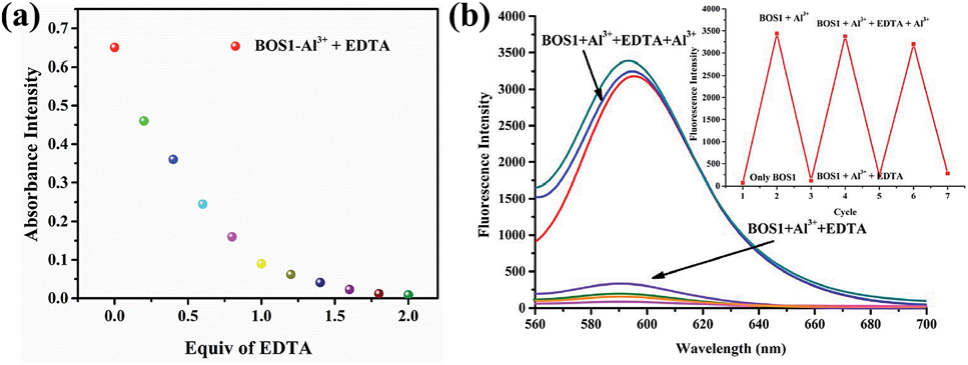
(a) Absorbance intensity changes at about 558 nm of BOS1 (10 μM) upon the addition of each equiv. of EDTA with the presence of Al^3+^ in ethanol–water (1 : 9, v/v, Tris–HCl, pH = 7.2) solution. (b) Fluorescence intensity of BOS1 + Al^3+^ as a function of EDTA concentration in ethanol–water (1 : 9, v/v, Tris–HCl, pH = 7.2) solution. Inset: central intensity changes of BOS1 for three cycles of the switching processes.

The binding reversibility of the probe BOS1 was also examined by the EDTA-adding experiments at room temperature. As illustrated in Fig. 9, the absorbance and fluorescence intensities rapidly decreased when EDTA was added to the Al^3+^–BOS1 system. Meanwhile, the color of the solutions changed from peach-red to colorless. When Al^3+^ ions were dropwise added into these systems again, the spectral signals were almost completely reproduced and the peach-red solutions appeared again. These reversible processes can be repeated several cycles without significant fluorescence changes (Fig. 9b, inset), indicating that the BOS probes are reversible fluorescence sensors toward Al^3+^.

### Cytotoxicity and fluorescence imaging

**Fig. 10:**
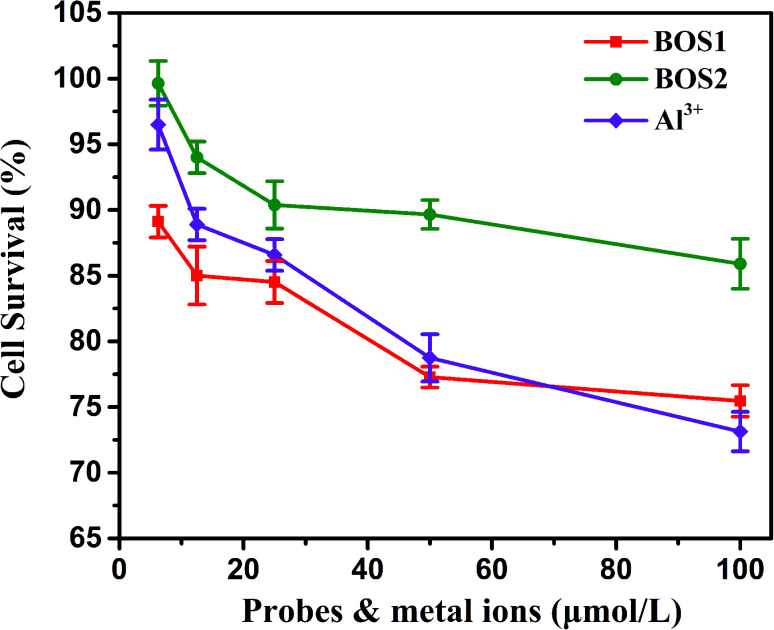
SGC-7901 living cells viability of BOS1, BOS2 and Al^3+^ quantified by the MTT assay (mean ± SD).

The MTT assays were performed to explore the cytotoxic effects of BOS1, BOS2 and Al^3+^ according to the reported method.^35^ The relevant data expressed as mean ± standard deviation were listed in Table S1† and the results were depicted in Fig. 10. The SGC-7901 living cells (human gastric carcinoma cells) viability remained 84.52%, 90.38% and 86.58% after the treatment of 25 μM probes BOS1, BOS2 and Al^3+^, respectively, which indicated that all of them were low cytotoxic to cells and suitable for bioimaging.

**Fig. 11:**
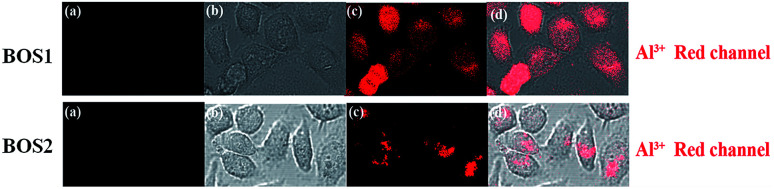
Probes BOS1 and BOS2 for Al(iii) fluorescence images in SGC-7901 living cells. Fluorescence images of SGC-7901 cells treated with probes (20 μM) in either the absence (a) or the presence (c) of 20 μM Al^3+^ ions for 1 h at 37 °C. (b) Bright-field image of cells shown in panel. (d) Overlay image of (b) and (c).

Fluorescence imaging experiments were conducted in the living cells to further demonstrate the practical applicability of the probes in biological samples.^45^Fig. 11 presented the fluorescence images of SGC-7901 cells recorded before and after the addition of Al^3+^. Apparently, free BOS1 and BOS2 probes showed no detectable fluorescence signals in living cells in the absence of Al^3+^ (Fig. 11a). By contrast, bright fluorescence signals were observed in living cells after incubation with Al^3+^ (Fig. 11c). Bright-field transmission images of cells treated with probes and target Al^3+^ ions showed that the cells were viable throughout the imaging experiments (Fig. 11b). The results suggested that probes BOS1/BOS2 possessed the capacity to readily penetrate the cell membrane and could be applied for *in vitro* imaging of Al^3+^ in living cells, and potentially *in vivo*.

## Conclusions

In summary, two novel rhodamine-based fluorescent probes BOS1 and BOS2 were designed and synthesized. Upon binding with Al^3+^, dramatic fluorescence and absorption enhancements were observed due to the formation of ring-opening of rhodamine species, showing distinct color changes and switch-on fluorescence. The probes displayed high selectivity, low detection limit, and fast response to Al^3+^ ions over other examined metal ions in ethanol–water system. Moreover, we have demonstrated their biological application by fluorescence imaging intracellular Al^3+^ in SGC-7901 living cells. We expect that the chemosensors would help to promote the studies of Al^3+^ in complex biological systems.

## Conflicts of interest

There are no conflicts to declare.

## Supplementary Material

RA-009-C8RA09850F-s001
