# Supplementary material for: New rhodamine B-based chromo-fluorogenic probes for highly selective detection of aluminium(iii) ions and their application in living cell imaging
Source: RSC Adv. 2019 Feb 19;9(11):6027–34. doi: 10.1039/c8ra09850f (PMC9060861; doi:10.1039/c8ra09850f)
Supplement: RA-009-C8RA09850F-s001 [file RA-009-C8RA09850F-s001.pdf]

Electronic Supplementary Material (ESI) for RSC Advances.
This journal is © The Royal Society of Chemistry 2019

Supporting Information

New rhodamine B-based chromo-fluorogenic probes for highly selective detection of aluminium(III) ion and their application in living cell imaging

Xin Leng,‡[a] Wenfeng Xu,‡[a] Chengfang Qiao,*[b] Xu Jia,[a] Ying Long,[a] and Bingqin Yang*[a]

[a] *Key Laboratory of Synthetic and Natural Functional Molecule Chemistry, Ministry of Education, College of Chemistry and Materials Science, Northwest University, Xi'an 710127, China.*

[b] *Shaanxi Key Laboratory of Comprehensive Utilization of Tailings Resources, College of Chemical Engineering and Modern Materials, Shangluo University, Shangluo 726000, China.*

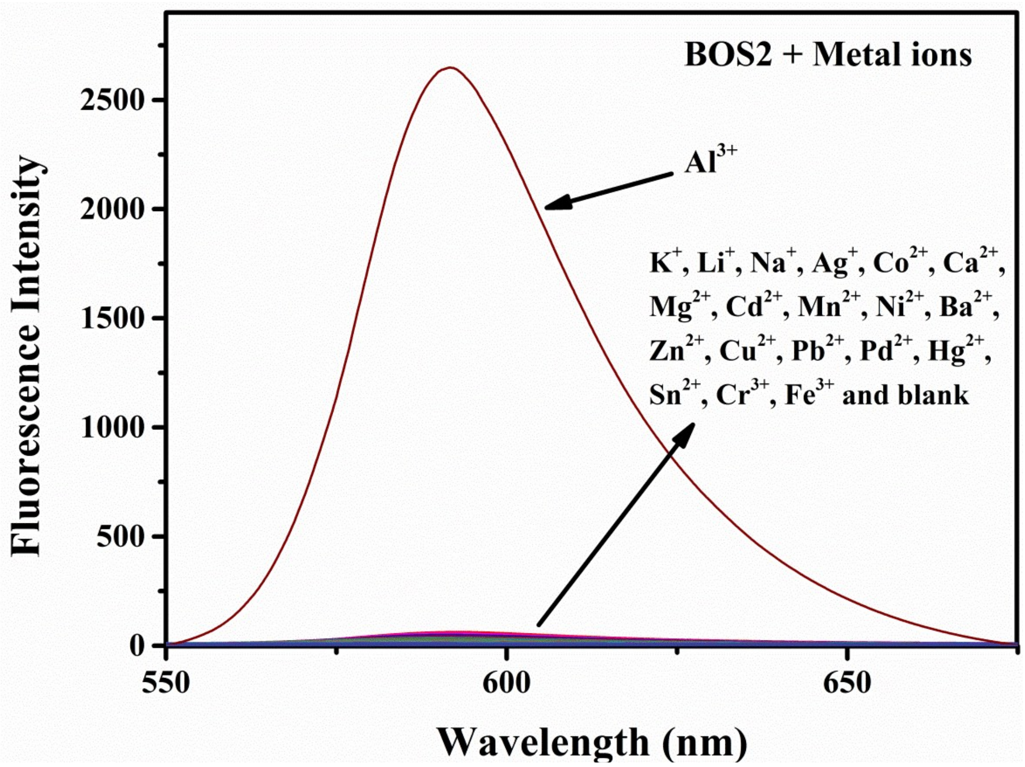

Fig. S1 Fluorescence spectra of **BOS2** (10 μM) in ethanol-water (1:9, v/v, Tris-HCl, pH =7.2) solution upon addition of various metal ions (10 μM).

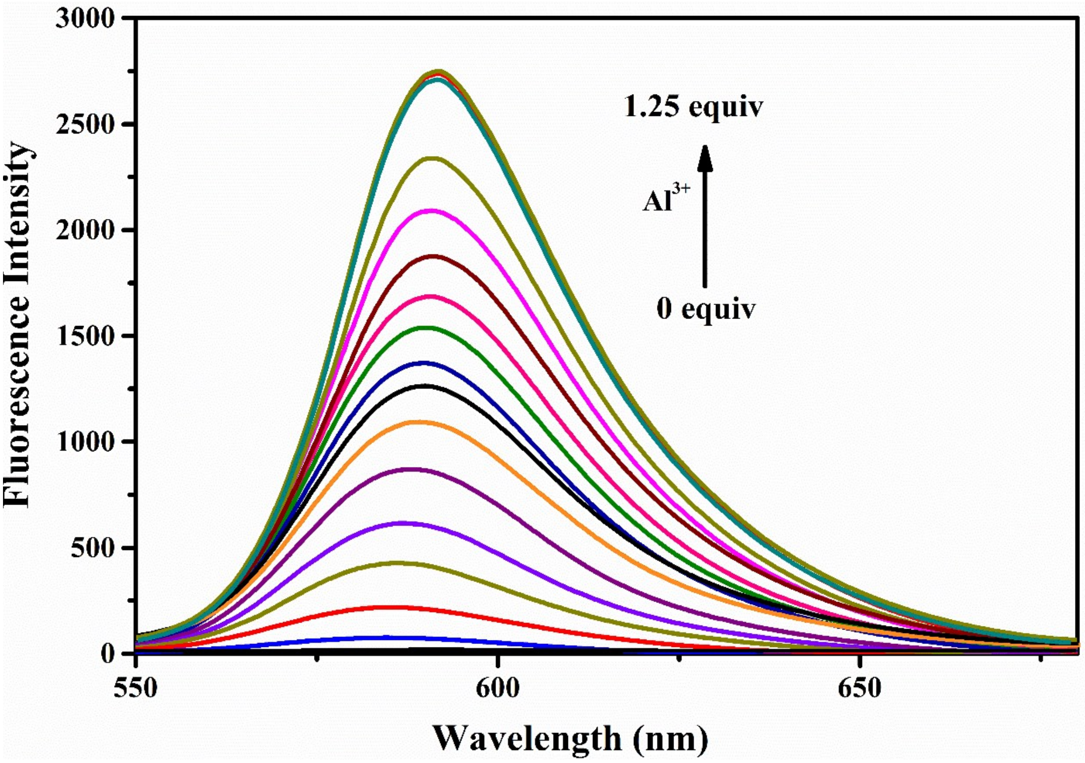

Fig. S2 Fluorescence spectra of **BOS2** (10 μM) with the addition of various concentrations of Al^{3+} ions (0–12.5 μM) in ethanol-water (1:9, v/v, Tris-HCl, pH =7.2) solution.

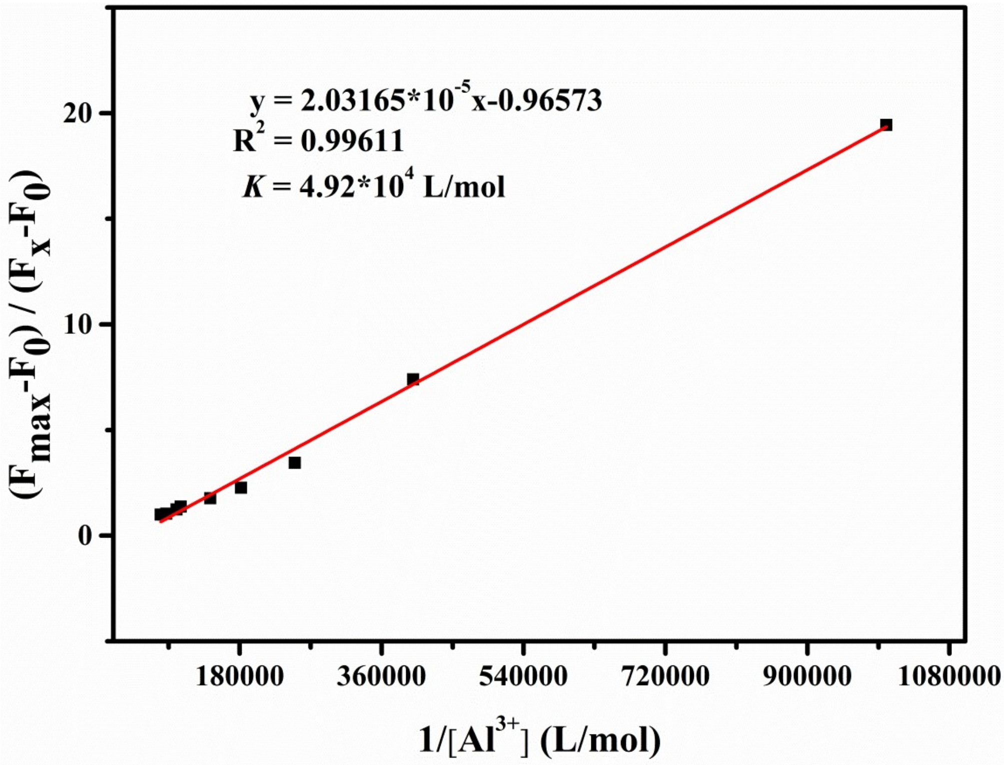

Fig. S3 Determination of binding constant of **BOS2** with Al^{3+} using Benesi–Hildebrand equation.

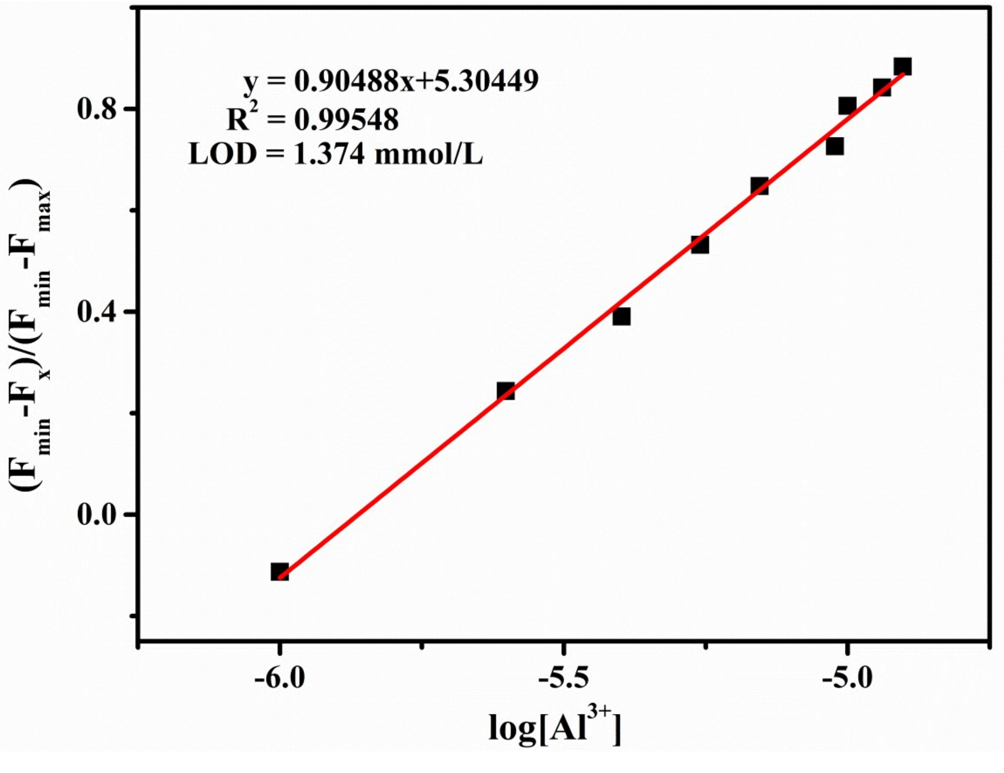

Fig. S4 The plot of $(F_{min}-F_x)/(F_{min}-F_{max})$ versus $\log[Al^{3+}]$ for the probe **BOS2**.

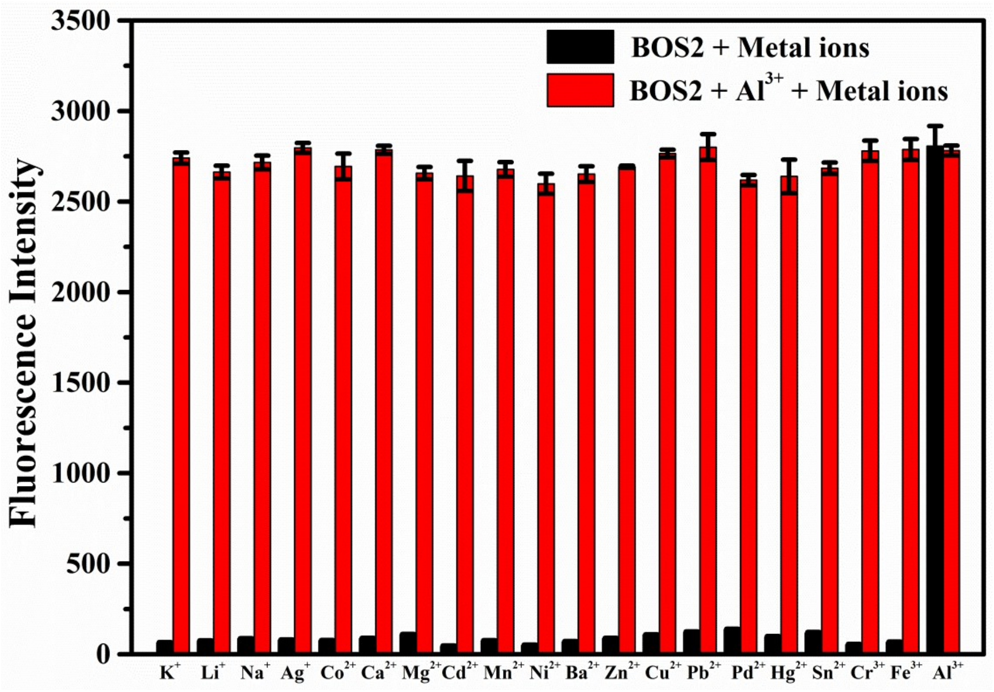

Fig. S5 Fluorescence intensity changes of **BOS2** (10 μM) upon the addition of various metal ions (10 μM) in the presence of Al^{3+} (10 μM) in ethanol-water (1:9, v/v, Tris-HCl, pH =7.2) solution. The black bars represent the fluorescence response of **BOS2** and metal ions. The red bars represent the subsequent addition of 10 μM Al^{3+} to the above solutions.

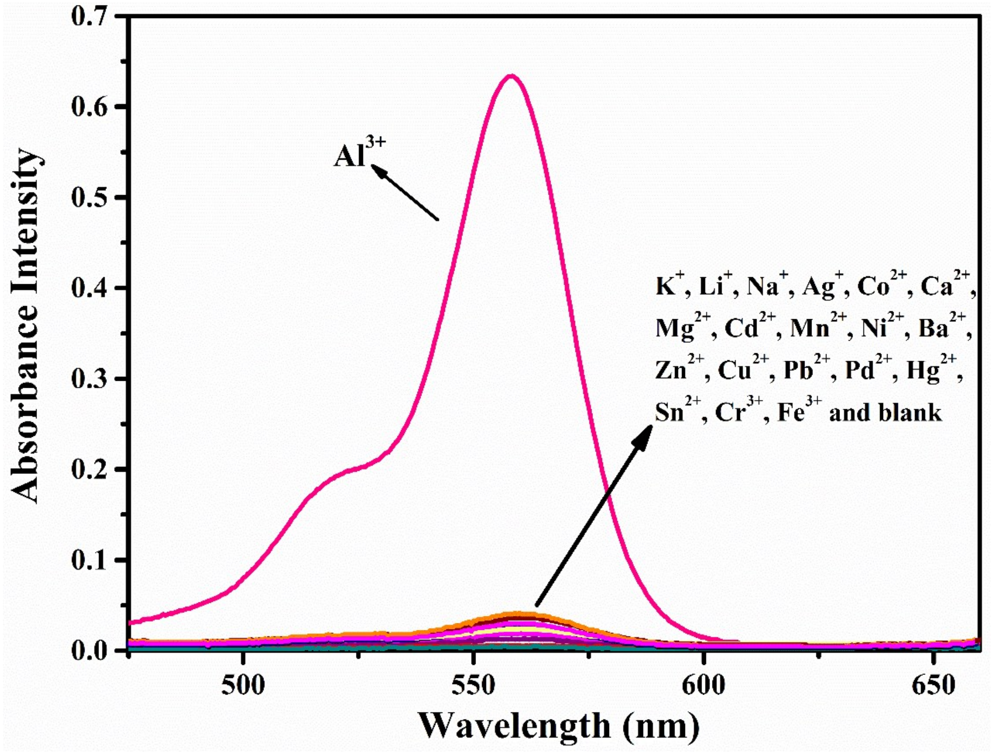

Fig. S6 UV–vis absorption spectra of **BOS2** (10 μM) in ethanol-water (1:9, v/v, Tris-HCl, pH =7.2) solution upon addition of various metal ions (10 μM).

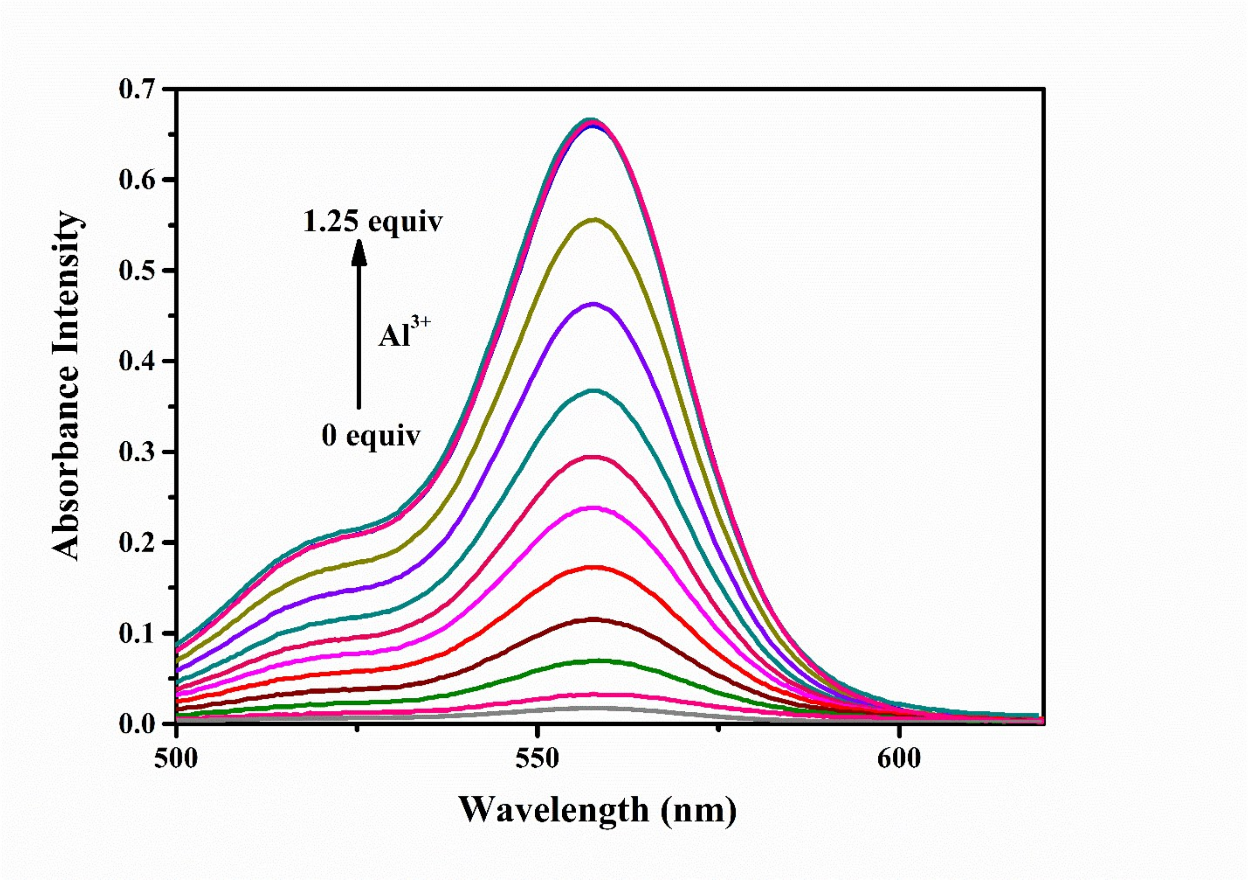

Fig. S7 UV–vis absorption spectra of **BOS2** (10 μM) with the addition of various concentrations of Al^{3+} ions (0–12.5 μM) in ethanol-water (1:9, v/v, Tris-HCl, pH =7.2) solution.

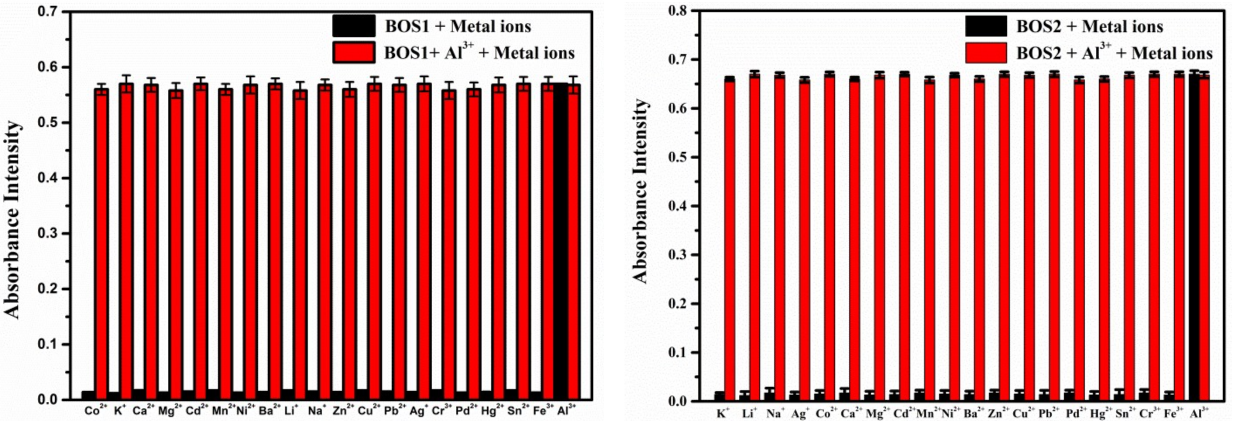

Fig. S8 UV–vis absorption intensity changes of 10 μM **BOS1** (left) and **BOS2** (right) upon the addition of various metal ions (10 μM) in the presence of Al^{3+} (10 μM) in ethanol-water (1:9, v/v, Tris-HCl, pH =7.2) solution. The black bars represent the absorption response of probes and metal ions. The red bars represent the subsequent addition of 10 μM Al^{3+} to the above solutions.

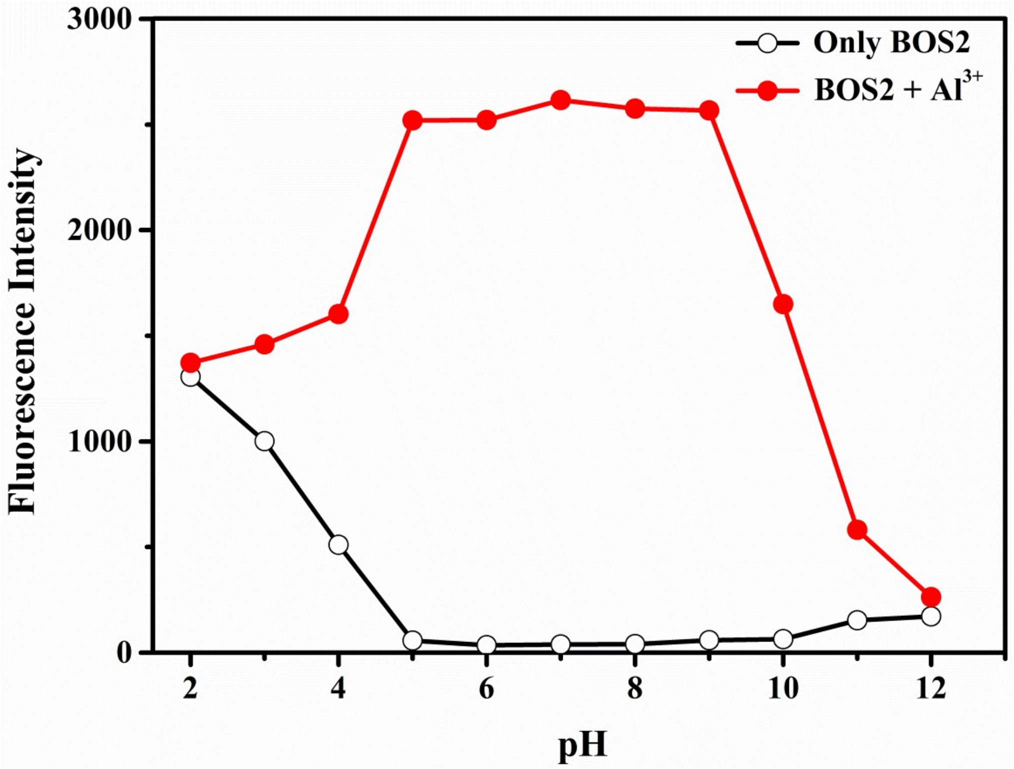

Fig. S9 Effects of pH on **BOS2** (10 μM) response to Al^{3+} (the pH of solution was adjusted by aqueous solution of NaOH (1 mol/L) and HCl (1 mol/L)).

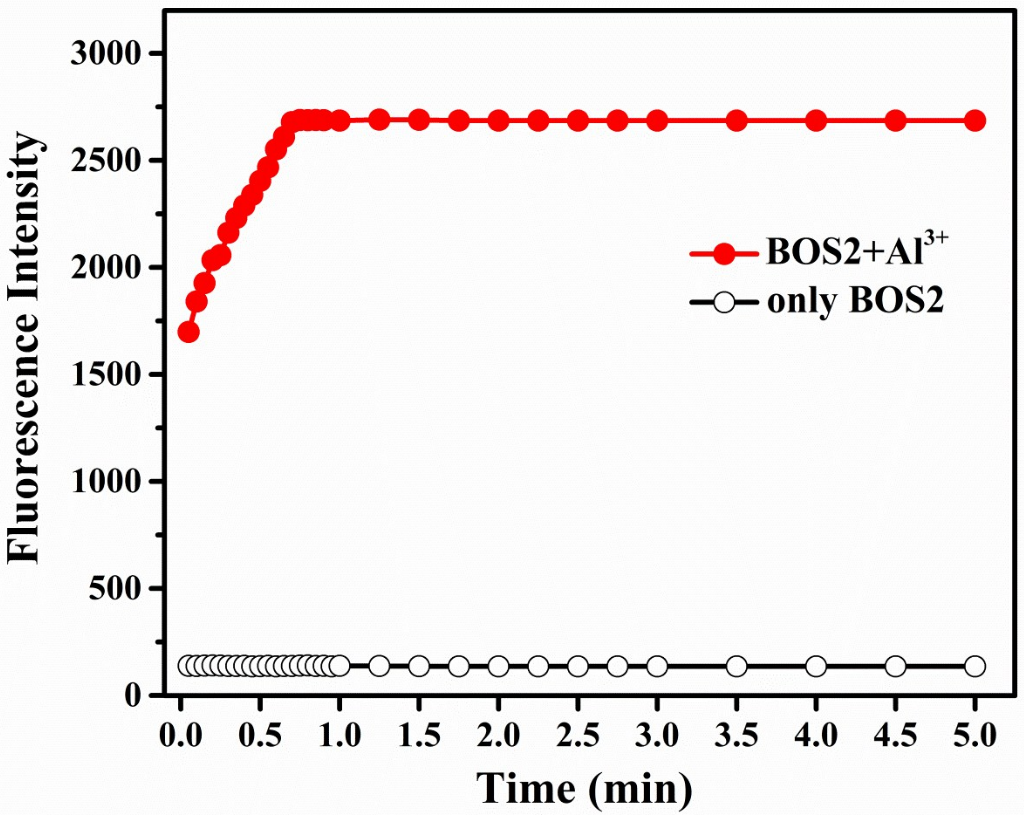

Fig. S10 Effects of time on **BOS2** (10 μM) response to Al^{3+} in ethanol-water (1:9, v/v, Tris-HCl, pH =7.2) solutions.

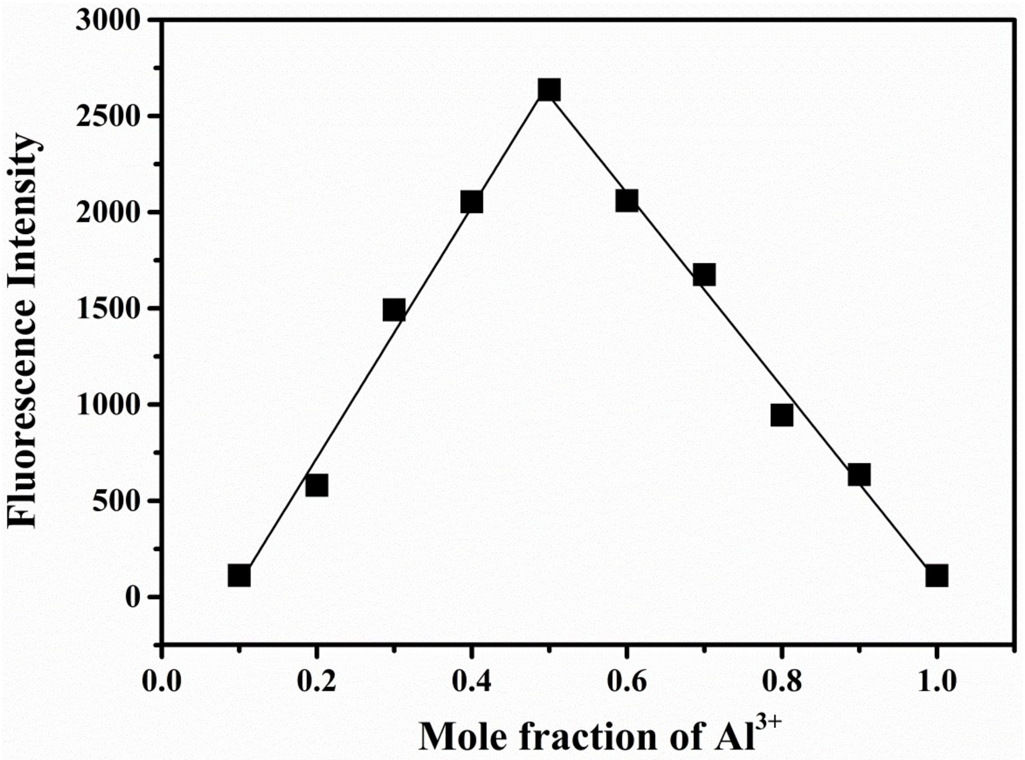

Fig. S11 Job's plot of **BOS2** and Al^{3+} (the total concentration was 10 μM).

Table S1 Cytotoxicity data of **BOS1**, **BOS2** and Al^{3+} (SGC 7901, 24h)

concentration	**BOS1**		**BOS2**		**Al^{3+}**	
(μmol/L)	Abs	Cell survival %	Abs	Cell survival %	Abs	Cell survival %
6.25	1.0094±0.1342	89.11	0.9187±0.0580	99.64	0.9566±0.0665	96.50
12.50	0.9627±0.0424	85.01	0.8667±0.0694	94.01	0.8813±0.0155	88.90
25.00	0.9573±0.1231	84.52	0.8333±0.0820	90.38	0.8583±0.0156	86.58
50.00	0.8753±0.0522	77.28	0.8267±0.0427	89.66	0.7807±0.0171	78.75
100.00	0.8547±0.0775	75.46	0.7920±0.0457	85.90	0.7250±0.0655	73.13
Blank	1.1327±0.0808	-------	0.9220±0.0125	-------	0.9913±0.0045	-------

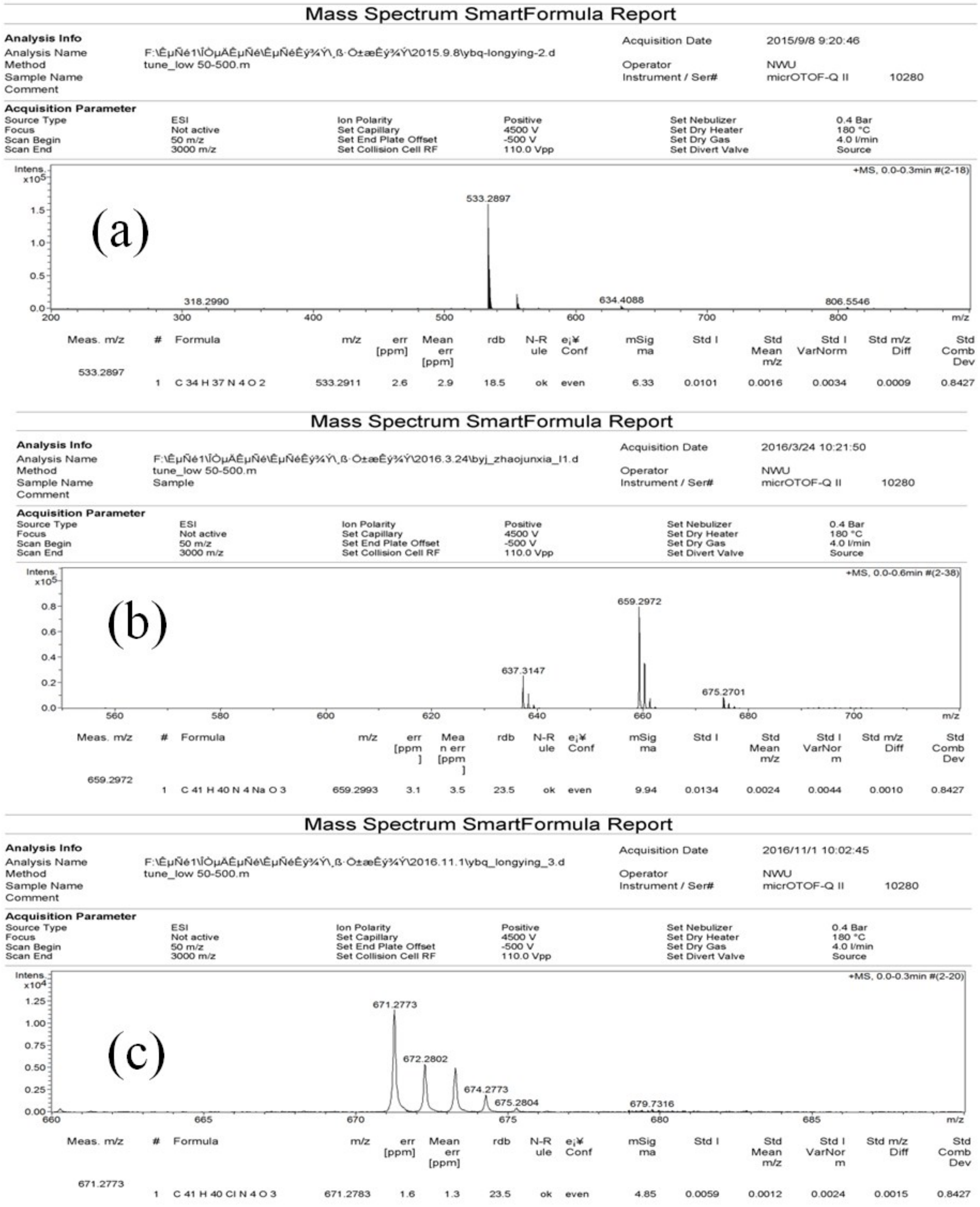

Fig. S12 ESI-MS spectra of **RBO** (a), **BOS1** (b), **BOS2** (c).

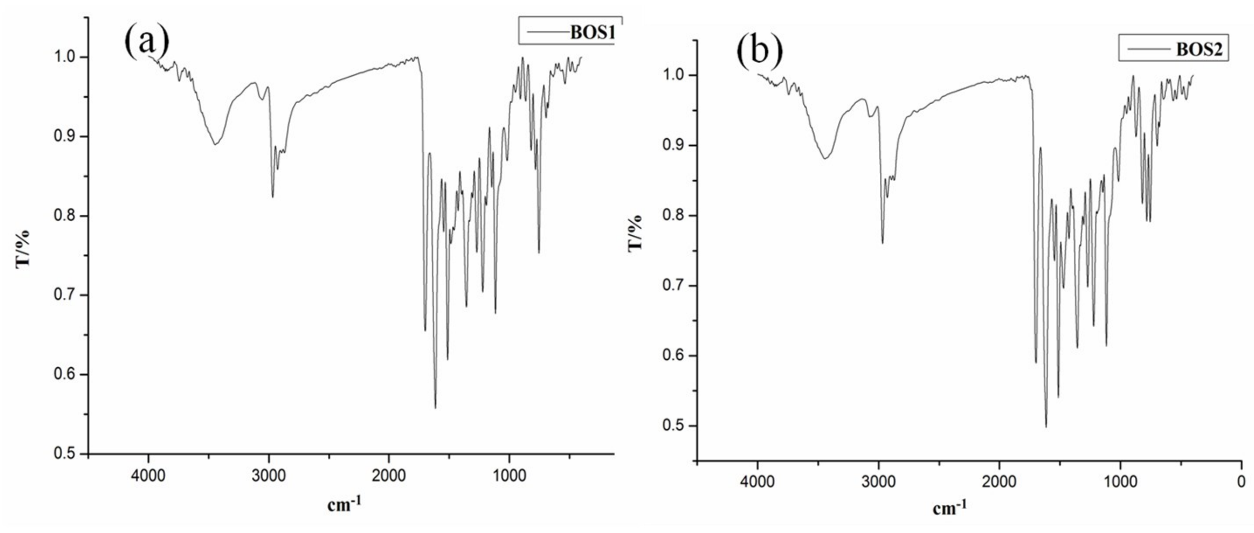

Fig. S13 The IR spectra of **BOS1** (a) and **BOS2** (b).

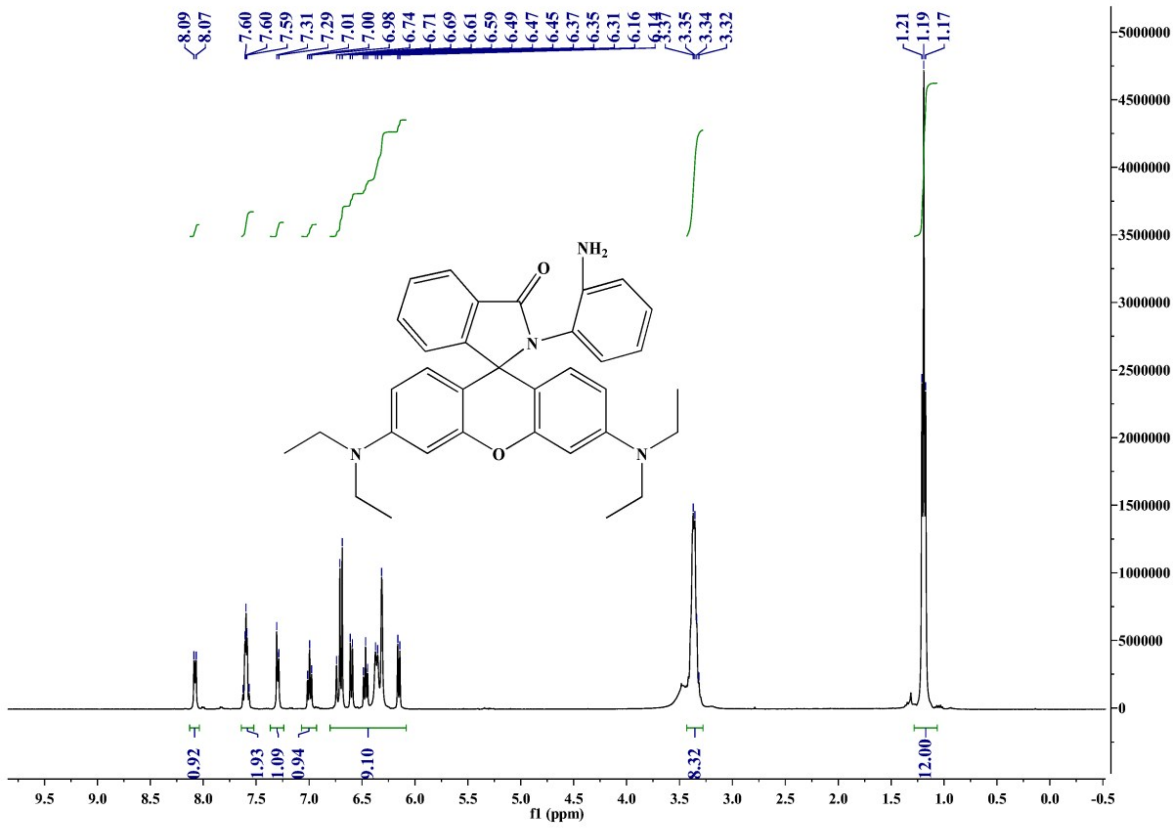

Fig. S14 The ^{1}H NMR spectrum of **RBO** in $CDCl_3$.

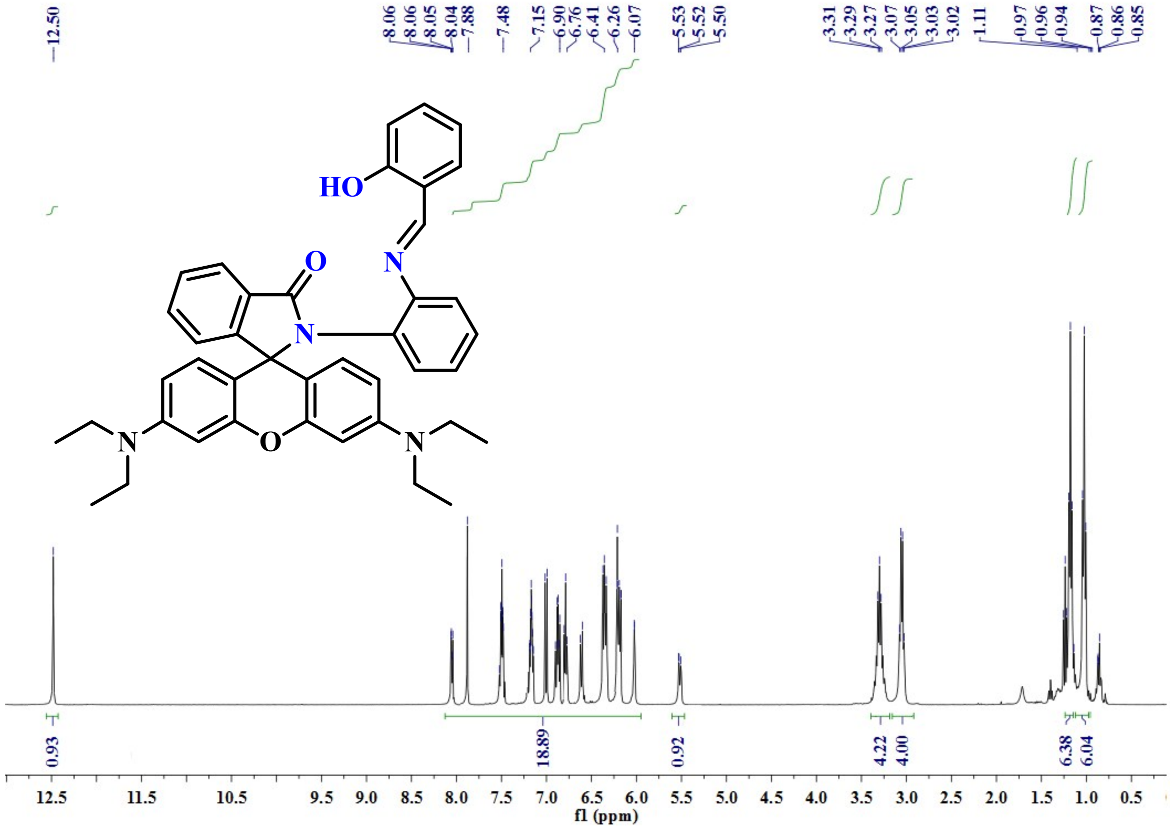

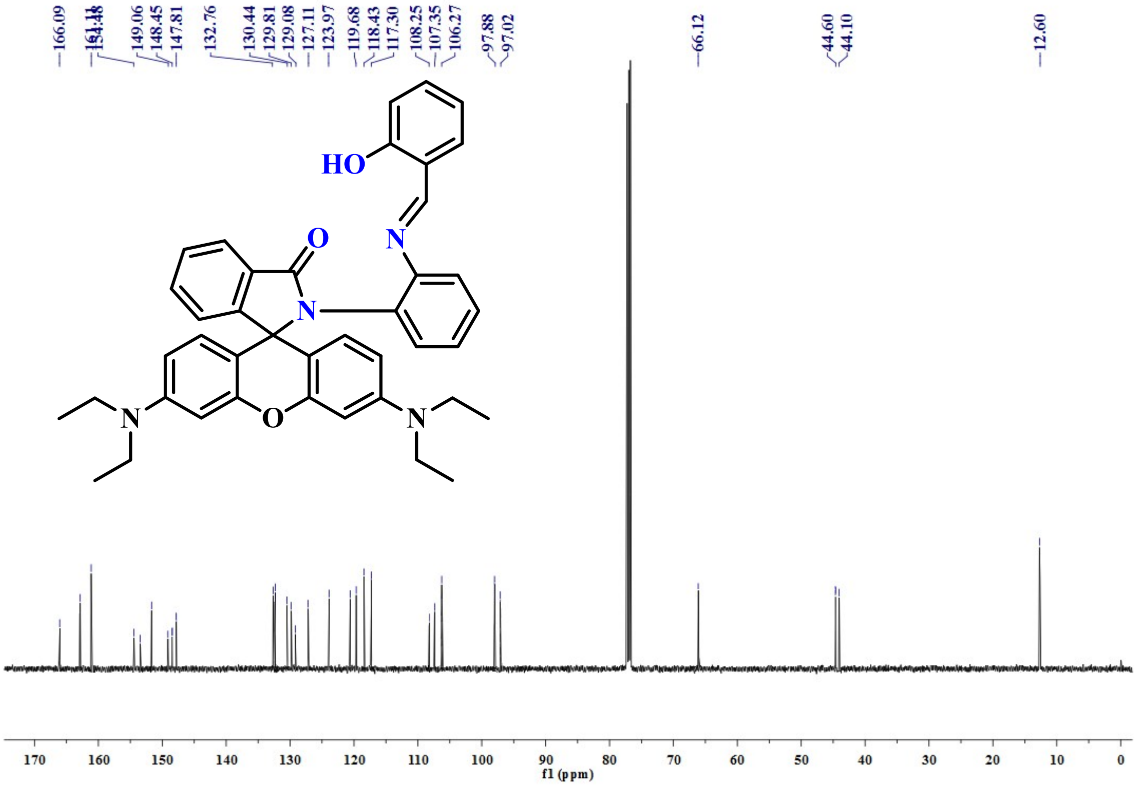

Fig. S15 The ^{1}H NMR (a) and ^{13}C NMR (b) spectra of **BOS1** in $CDCl_3$.

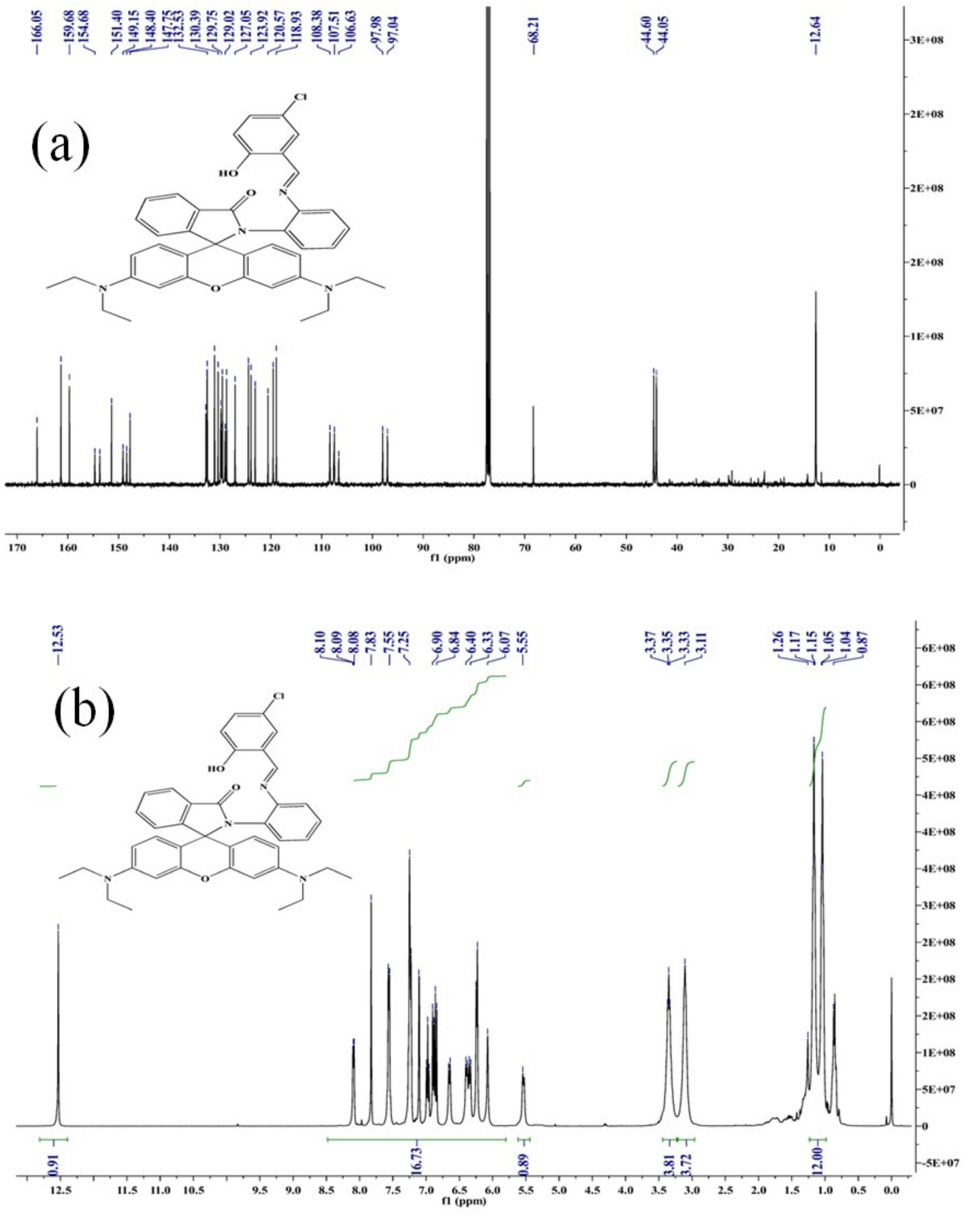

Fig. S16 The ^{13}C NMR (a) and ^{1}H NMR (b) spectra of **BOS2** in $CDCl_3$.
